# Biopsy-Proven Renal-Limited Thrombotic Microangiopathy in a Heart Transplant Recipient

**DOI:** 10.1016/j.ekir.2023.03.004

**Published:** 2023-03-12

**Authors:** Joyita Bharati, Syed Haider, Simon W. Maybaum, Kenar D. Jhaveri, Vanesa Bijol, Nupur N. Uppal

**Affiliations:** 1 Division of Kidney Diseases and Hypertension, Donald and Barbara Zucker School of Medicine, Hofstra University, Great Neck, New York, USA; 2 Glomerular Center at Northwell, Donald and Barbara Zucker School of Medicine, Hofstra University, Great Neck, New York, USA; 3 Department of Cardiology, Donald and Barbara Zucker School of Medicine, Manhasset, New York, USA; 4 Department of Pathology, Donald and Barbara Zucker School of Medicine, Manhasset, New York, USA

**To the Editor:**

Thrombotic microangiopathy (TMA) is known to occur in the setting of solid organ transplantation.^1^ TMA after heart transplant is rare and usually described as hemolytic uremic syndrome comprising of acute kidney injury (AKI) and microangiopathic hemolytic anemia.^2^, ^3^, ^4^, ^5^ We report a case of AKI without microangiopathic hemolytic anemia caused by TMA following orthotopic heart transplant. To the best of our knowledge, this is the first report to illustrate biopsy-proven renal-limited TMA after heart transplant.

A 58-year-old male underwent orthotopic heart transplant for nonischemic cardiomyopathy in January 2021. On day 3 after transplant, he developed pericardial effusion and oliguric AKI requiring hemodialysis. Kidney function recovered over the next week and the patient was discharged with a serum creatinine of 2.6 mg/dl. Tacrolimus, mycophenolate mofetil, and steroids were used for maintenance immunosuppression. The patient was readmitted 2 months following transplant with gastrointestinal bleeding when his serum creatinine increased to 3.2 mg/dl. Esophagoduodenoscopy and colonoscopy revealed gastritis and uncomplicated diverticulosis, respectively. Stool polymerase chain reactionfor Shiga toxin was negative. He was noted to have new-onset proteinuria of 2 g/d. Although the provisional diagnosis for AKI was acute tubular necrosis secondary to hypovolemia and/or acute calcineurin inhibitor toxicity, kidney biopsy revealed acute and chronic TMA (Figure 1). There was no evidence of microangiopathic hemolytic anemia (hemoglobin: 8.8 g/dl, platelets: 241,000 per mm^3^, lactate dehydrogenase: 157 IU/l, haptoglobin: 41 mg/dl, peripheral smear: no schistocytes). Complement testing revealed normal complement (C3, C4) levels, elevated SC5b-9 complex (374 ng/ml), and a heterozygous complement factor H receptor 5 gene mutation (of unknown significance). Viral causes of TMA were ruled out. A possibility of complement-mediated TMA triggered by tacrolimus or transplantation *per se* was kept. Tacrolimus was replaced with everolimus and weekly eculizumab (900 mg) was started. However, the patient developed respiratory failure secondary to everolimus-induced pneumonitis and his kidney function worsened to dialysis dependency. Everolimus was switched to cyclosporine and eculizumab was continued. The patient maintained cardiac allograft function without any significant rejection.

**Figure 1:**
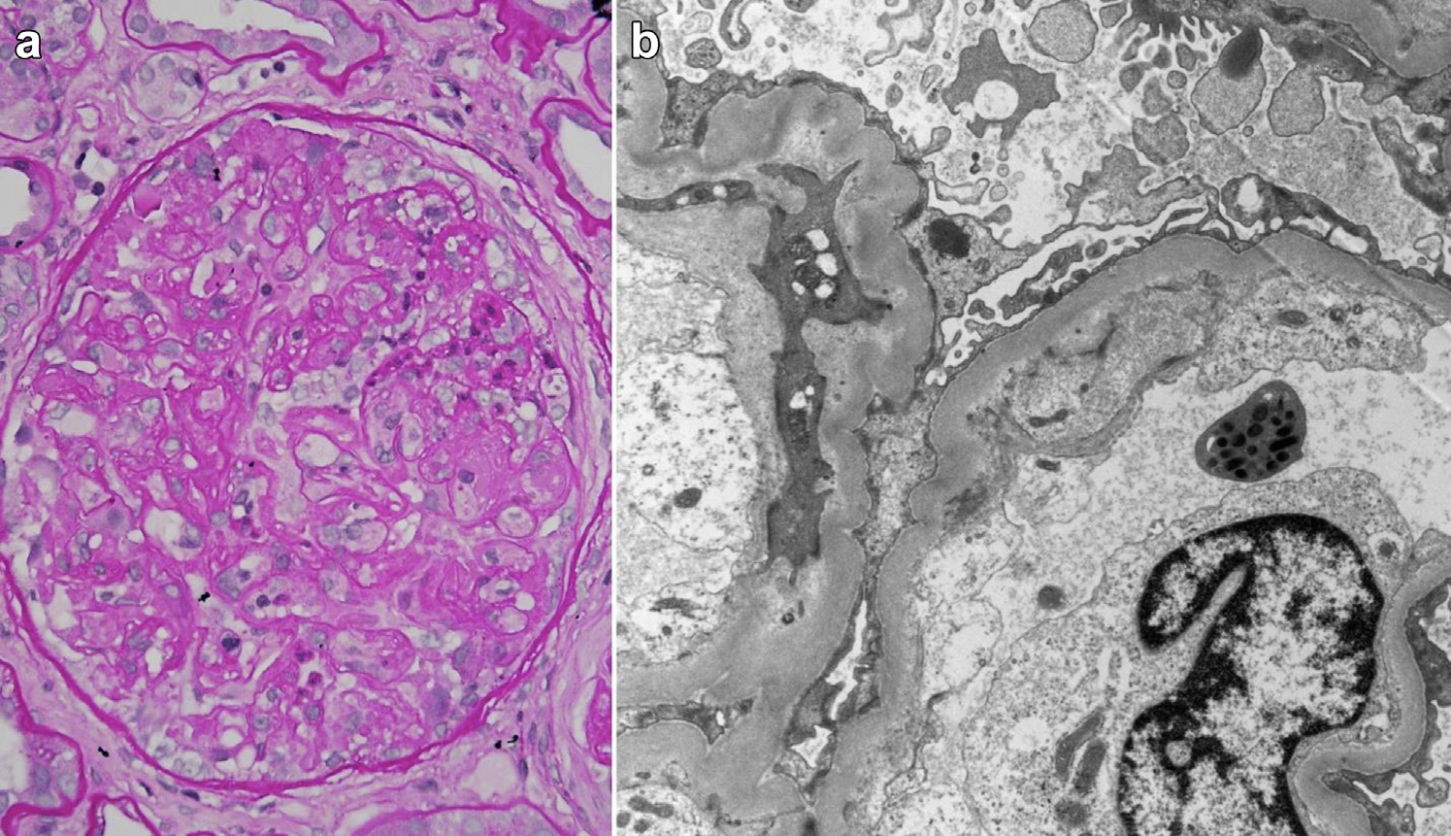
(a) Kidney biopsy findings of acute and chronic thrombotic microangiopathy: segmental thrombosis of the glomerular tuft (left, Periodic acid-Schiff, 400×), (b) Glomerular capillary wall remodeling with cellular projections and new basement membrane formation under the swollen endothelium (electron micrograph, original magnification 6700×).

TMA can present as AKI following orthotopic heart transplant in the absence of hematological findings. Kidney biopsy in a case of kidney dysfunction following heart transplant is pivotal and should be given early consideration.

## Disclosure

All the authors declared no competing interests.
